# Navigating the orphan medicinal product designation: Evidence requirements for gene therapies in Europe

**DOI:** 10.1016/j.ymthe.2024.10.015

**Published:** 2024-10-28

**Authors:** Gloria M. Palomo, Tomas Pose-Boirazian, Frauke Naumann-Winter, Enrico Costa, Dinah M. Duarte, Maria E. Kalland, Eva Malikova, Darius Matusevicius, Dinko Vitezic, Kristina Larsson, Armando Magrelli, Violeta Stoyanova-Beninska, Segundo Mariz

**Affiliations:** 1 Agencia Española de Medicamentos y Productos Sanitarios, Calle Campezo 1 Edificio 8, 28022 Madrid, Spain; 2 Orphan Medicines Office, European Medicines Agency, Domenico Scarlattilaan 6, 1083 HS Amsterdam, the Netherlands; 3 Bundesinstitut für Arzneimittel und Medizinprodukte, Kurt-Georg-Kiesinger-Allee 3, 53175 Bonn, Germany; 4 Agenzia Italiana del Farmaco, Via del Tritone 181, 00187 Rome, Italy; 5 INFARMED - National Authority of Medicines and Health Products, I.P., 1749-004 Lisbon, Portugal; 6 Universidade de Lisboa, Faculdade de Farmácia, 1649-003 Lisbon, Portugal; 7 Norwegian Medical Products Agency, Grensesvingen 26, 0663 Oslo, Norway; 8 Department of Pharmacology and Toxicology, Comenius University, 832 32 Bratislava, Slovakia; 9 Läkemedelsverket, Dag Hammarskjölds väg 42, 75237 Uppsala, Sweden; 10 Rijeka University School of Medicine and University Hospital Centre Rijeka, Braće Branchetta 20, 51000 Rijeka, Croatia; 11 National Center for Drug Research and Evaluation, Istituto Superiore di Sanità, Viale Regina Elena 299, 00161 Rome, Italy; 12 Committee for Orphan Medicinal Products, European Medicines Agency, Domenico Scarlattilaan 6, 1083 HS, Amsterdam, the Netherlands

**Keywords:** Gene therapy, orphan medicinal product, medical plausibility, significant benefit, orphan designation, rare conditions, clinically relevant advantage, advance therapy medicinal products, animal models, clinical data

## Abstract

To provide insight into regulatory decision-making at the time of granting initial orphan designation by the Committee for Orphan Medicinal Products, we have conducted a retrospective analysis for viral vector-mediated gene therapies in rare non-oncological conditions with respect to the data provided to support the criteria to be met in successful applications. We found that a high proportion of non-clinical *in vivo* data was used for gene therapies, indicating earlier submissions of products that are at the stage of preclinical research and not in clinical development. Clinical data were submitted in only 13% of the applications, containing preliminary results derived from early-stage clinical trials in few patients. Mouse models were used in the majority of the submissions to generate meaningful non-clinical *in vivo* data highlighting their utility for proof-of-concept studies, and half of the applications containing non-clinical data generated results based solely on surrogate endpoints. The criterion of significant benefit was applicable in 54% of the submissions, which indicates that sponsors are focusing gene therapy development in areas of high unmet medical need, particularly where there are no authorized medicines available.

## Graphical abstract

**Figure:**
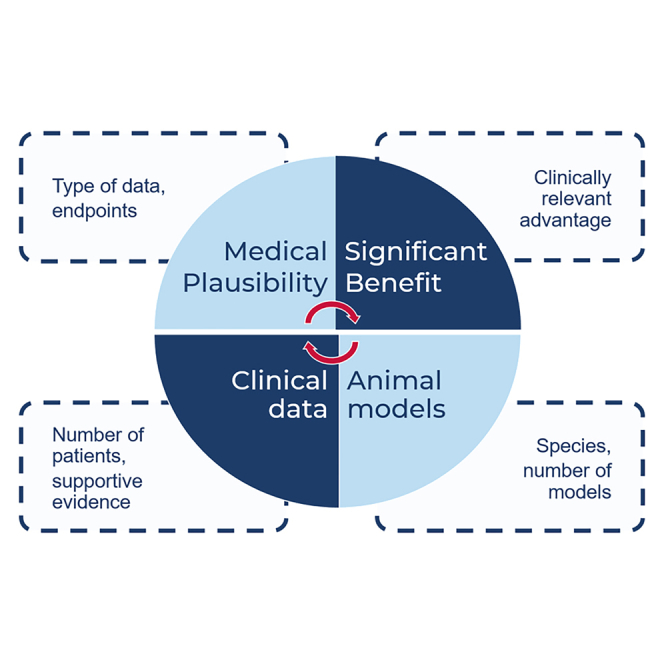

## Teaser

Palomo, Mariz, and colleagues describe the data submitted in successful applications for orphan designation for viral vector-based gene therapy products in rare non-oncological conditions. A description of the particularities and acceptance of the data to support two important orphan designation criteria might benefit product developers in future applications.

## Introduction

For patients with diseases caused by known gene mutations, gene therapies offer the potential to restore normal gene and protein function and potentially cure the underlying condition.^1^ Most of these diseases affect a limited number of patients, and therefore the pair product/condition might be amenable for orphan designation (OD) in the European Union (EU), if compliant with criteria as defined in the EU Orphan Regulation.^2^^,^^3^ To date, most gene therapy medicinal products that received a marketing authorization (MA) in the EU have also received an OD, except Imlygic, which was developed for the non-rare condition melanoma.^4^ Securing an OD for these medicinal products in Europe can be an important step, as several pre- and post-authorization incentives are linked to the designation.^5^^,^^6^ The Committee for Orphan Medicinal Products (COMP) is the European Medicines Agency (EMA) scientific committee responsible for the assessment of the OD applications at two stages, during product development (initial OD) and at the time of MA or extension of indication (maintenance of the OD), following its main role in fostering the development of medicines to tackle rare diseases.

An OD submission generally includes information in several domains to ascertain that both the disease and the product are compliant with regulatory requirements.^7^ The proposed orphan condition should be justified as chronically debilitating and/or life threatening and also rare, which requires it to have a prevalence below 5 in 10,000 persons in the EU. The latter criterion should be justified through sources available in the public domain. For an initial OD application, data with the proposed medicinal product must be submitted to support the hypothesis of its potential effectiveness in the target orphan condition. This requirement is commonly known as “medical plausibility” (MP) or the “intention to diagnose, prevent, or treat.”^8^ A unique feature of the Orphan Regulation in Europe is the criterion of significant benefit (SB), which applies when authorized medicines for the orphan condition exist in the EU.^9^^,^^10^ In this case, the sponsor is required to submit data with their product, in the target orphan condition, supporting the assumption of a potential additional therapeutic benefit for affected patients over the authorized medicinal products, commonly referred to as satisfactory methods of treatment. SB is described in the legislation as a clinically relevant advantage (CRA) and/or a major contribution to patient care (MCPC).^11^ At the stage of initial OD, with products often early in development, a well-justified assumption of SB using preliminary non-clinical or clinical data can be accepted. A substantial proportion of initial OD submissions (more than two-thirds) requires a justification of SB as the candidate products are being developed in orphan conditions with satisfactory methods of treatment.^12^

The COMP, as part of its role in promoting the development and placement on the market of medicines for rare diseases, launched in late 2021 an initiative to study the rising number of OD submissions seen in recent years pertaining to advanced therapy medicinal products (ATMPs), with the aim of guiding future developments.^4^ The focus of this revision was primarily on gene therapy products that use viral-derived vectors as a delivery system for the treatment of non-oncological conditions. These represent the largest group among ATMP products; other types of ATMPs not belonging to this group are not included in the analysis. In the present review, we examined the data submitted to support the criteria of MP and the justification of SB, as a continuation of the previously described product characteristics and classification. The aim of this retrospective analysis was to provide insights into the regulatory decision-making process, more specifically, a positive outcome on OD related to sufficient supportive evidence for MP and SB.

## Results

### Medical plausibility

**Figure 1:**
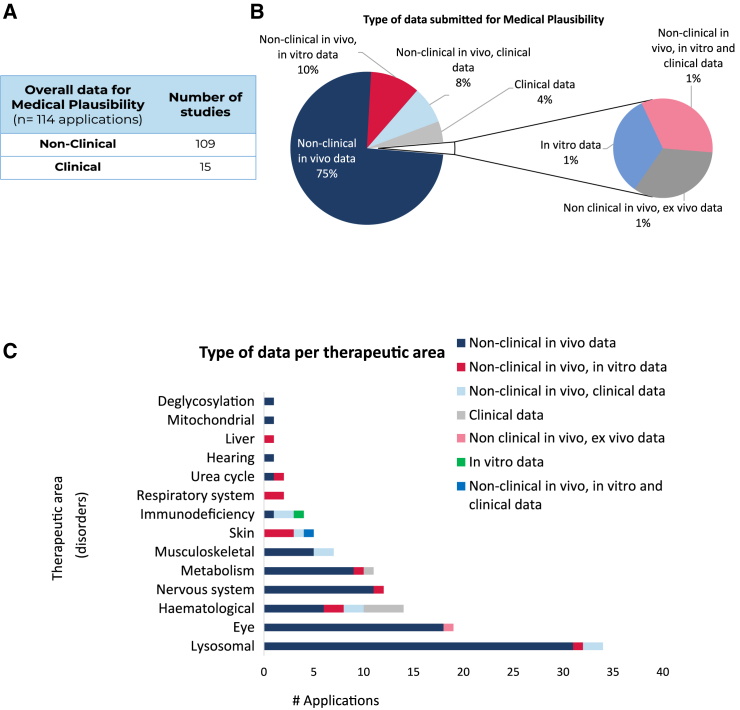
Analysis of the data provided in support of the medical plausibility criterion for orphan medicinal product designation (A) Overall distribution in two categories of the studies provided by the sponsors: non-clinical data and clinical data. (B) In-depth analysis of the type of data submitted. The studies were classified according to the following categories: non-clinical *in vivo*, non-clinical *in vitro*, non-clinical *ex vivo*, and clinical data. (C) Distribution of the type of data provided following classification into therapeutic areas. Total number of applications, *n* = 114.

Of the 114 submissions, non-clinical *in vivo* data were provided in 109, while only 15 contained clinical data (Figure 1A). Given that those two categories coexist in some applications, a detailed analysis of the data provided to support MP identified different combinations regarding data submissions. Seventy-five percent of applications were supported by non-clinical *in vivo* data only, followed by a combination of non-clinical *in vitro* and *in vivo* data (10%), non-clinical *in vivo* with clinical data (8%), and 4% based exclusively on clinical data. Overall, 12% contained *in vitro* data; however, one submission had only *in vitro* data (1%) (Figure 1B). The distribution of these combinations following categorization into therapeutic areas revealed certain particularities (Figure 1C). The areas with the majority of the submissions based on non-clinical *in vivo* studies were lysosomal, eye, nervous, and musculoskeletal disorders (of note, in one application for eye disorders, the non-clinical *in vivo* data were supported with *ex vivo* data in porcine retinas). Other therapeutic areas such as skin, respiratory system, and liver disorders often contained a combination of *in vitro* and non-clinical *in vivo* data. Five submissions contained only clinical data: four were for hematological disorders, and one was for metabolism disorders. *In vitro* data were submitted alone in a condition in the group called “immunodeficiency disorders.”

### Complexity of the non-clinical data

**Figure 2:**
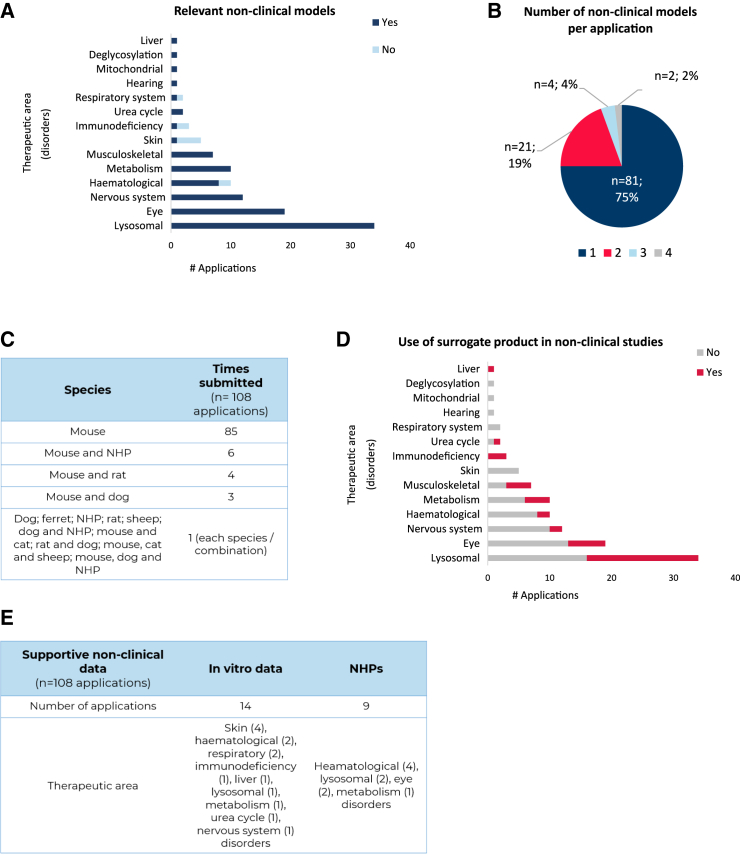
Complexity of the non-clinical *in vivo* data (A) Distribution of the studies conducted in relevant non-clinical models following the therapeutic areas. (B) Overall analysis of the number of non-clinical models per application. (C) Species used to generate the non-clinical *in vivo* data (single species and combinations). The number of times that each species was submitted was recorded. (D) Classification per therapeutic area showing the use of surrogate products in the non-clinical *in vivo* studies. (E) Analysis of the supportive non-clinical data, either *in vitro* data or use of non-human primates (NHPs), and their distribution per therapeutic area. Total number of applications containing non-clinical *in vivo* data, *n* = 108.

Overall, the animal models used to support the MP criterion for OD are cited in the literature and are therefore well-known and relevant as they resemble the target orphan human condition.^13^^,^^14^ In our analysis, relevant non-clinical *in vivo* models of the target condition were used in all the therapeutic areas (Figure 2A), with several exceptions. In hematological, skin, immunodeficiency, and respiratory system disorders, some applications contained data in non-relevant *in vivo* models of the condition (9 out of a total of 108 applications with non-clinical *in vivo* data). Of note, for the products intended for skin disorders, 4 out of 5 used non-relevant animal models, and all of them tested either topical, intradermal, or graft-in-skin as it was the intended route of administration for the final product, which requires an animal model that allows engraftment. Seventy-five percent of ODs provided data in only one animal model per application (Figure 2B). This was followed by 19% that used data in two different models, and only 4% and 2% used 3 and 4 different models, respectively. Mice were by far the species most used for non-clinical *in vivo* studies, with data presented in 101 submissions (Figure 2C). Other species used were dog (7 submissions in total) and rats (6). Rarely used models involved cats (2), sheep (2), and ferrets (1).

The use of a surrogate product in the setting of gene therapies was high (Figure 2D). Disorders where this approach was used were liver, urea cycle, immunodeficiency, musculoskeletal, metabolic, hematological, nervous system, eye, and lysosomal. These types of products were not used in skin, deglycosylation, mitochondrial, hearing, and respiratory disorders.

Supportive *in vitro* data were included in 14 applications, including patients’ derived cells of diverse tissue origin (e.g., fibroblasts, hepatic cell lines, keratinocytes), more frequently used in support of the proposed mechanism of action, and continuous cell lines, which were less frequently used, to assess efficiency of transduction and protein expression levels (Figure 2E). Only one application was sustained entirely with *in vitro* data in patients’ cells (for immunodeficiency disorders), since no relevant model of the disease was available at the time the application was submitted.

**Figure 3:**
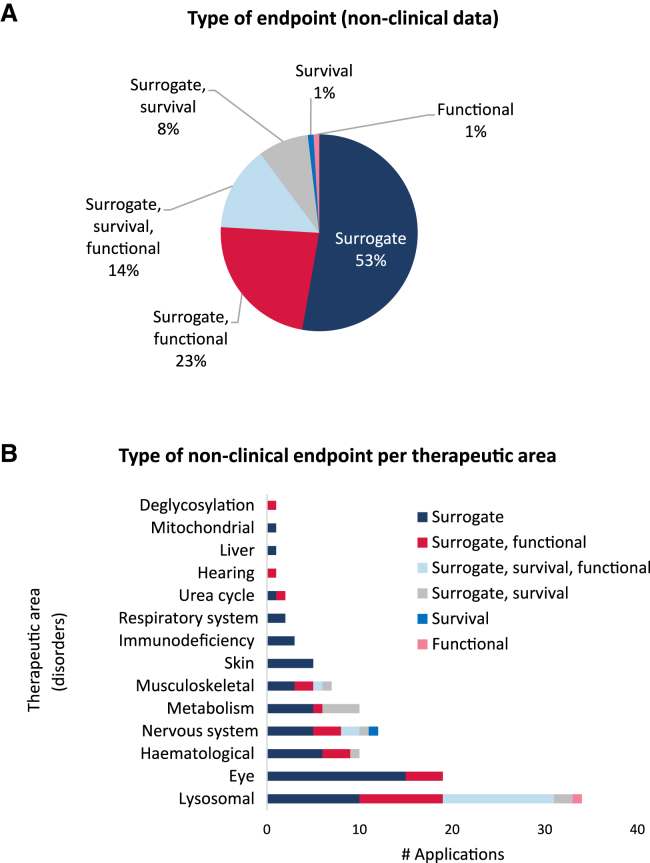
Analysis of the endpoints assessed in the non-clinical *in vivo* studies submitted (A) Distribution of the different categories (surrogate, functional, and survival) either alone or in combination. (B) Distribution of the endpoints recorded, alone or in combination, per therapeutic area. Total number of applications containing non-clinical *in vivo* data, *n* = 108.

It was noted that almost all submissions (106 of 108 based on non-clinical *in vivo* data) had surrogate endpoints to measure efficacy (Figure 3A). We were interested in understanding what type of combinations involving surrogate, functional, and survival endpoints were most used in the applications submitted. A combination of surrogate and functional endpoints accounted for a total of 25 submissions (23%). A total of 15 applications contained non-clinical data comprising surrogate, functional, and survival endpoints (14%), and only 9 were based in surrogate endpoints and survival data (8%). The endpoint data were examined per therapeutic area (Figure 3B). Areas that only had surrogate endpoints involved those of the skin, immunodeficiencies, respiratory system, liver, and mitochondria disorders, although some of the latter areas were less represented in the therapeutic area distribution. The combination surrogate, survival, and functional was noted in conditions classified as lysosomal, nervous system, and musculoskeletal disorders. Survival as an endpoint was reported in lysosomal, hematological, nervous system, metabolism and musculoskeletal disorders. Only one application was based in functional data (in a lysosomal disease), and one for a neurological disease was based solely on survival data.

### Clinical data

**Figure 4:**
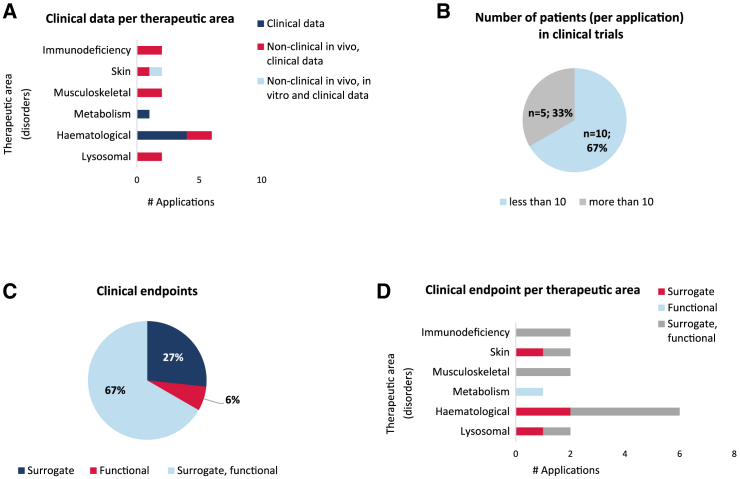
Complexity of the clinical data (A) Distribution of the clinical data submitted in support of the medical plausibility criterion for orphan medicinal product designation, alone or in combination with non-clinical data, and classified according to the therapeutic areas. (B) The number of patients per clinical trial was classified in two categories: less than 10 and more than 10. The distribution of the applications for orphan designation following these two categories was recorded. (C) Analysis of the endpoints studied in the clinical data provided. The endpoints were classified either as surrogate or functional, and their use alone or in combination was registered. (D) Distribution of the clinical endpoints per therapeutic area. Total number of applications containing clinical data, *n* = 15.

Fifteen ODs (or 13% of the total) had preliminary clinical trial data in patients with the condition (Figure 4A). Five of these 15 only had clinical data (4 for hematological and 1 for metabolism disorders) to support the MP. The remaining ODs had data from both non-clinical and clinical studies, where the non-clinical studies were used to support the limited preliminary clinical data. Clinical and non-clinical *in vivo* data were used in ODs for hematological, lysosomal, musculoskeletal, skin, and immunodeficiency disorders. The number of patients included in the trials varied and was grouped into studies that had fewer than 10 patients and those that had more than 10. Most submissions (10 out of 15) had clinical data with 10 or fewer patients enrolled (Figure 4B).

Most submissions included clinical data that used endpoints which entailed a combination of surrogate and functional (Figure 4C). A limited number of applications (4 of 15) used a surrogate endpoint only, which were noted in lysosomal, hematological, and skin disorders (Figure 4D).

### Significant benefit

**Figure 5:**
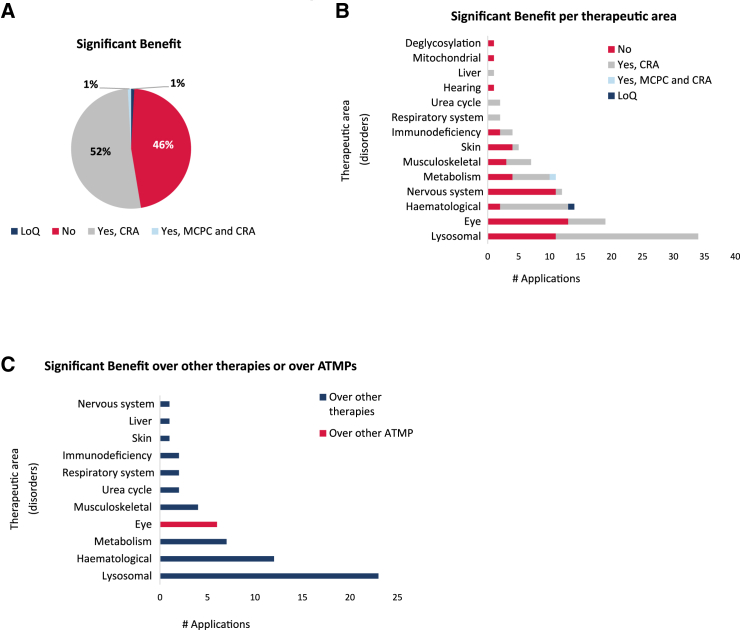
Analysis of the significant benefit criterion for orphan medicinal product designation (A) Distribution of the applications regarding the claim of significant benefit (SB). The applications were stratified in four different categories: no claim of SB, claim of SB based on clinically relevant advantage (CRA), claim of SB based on CRA and major contribution to patient care (MCPC), and not assessed beyond the list of questions (LoQ). (B) Distribution of the SB claims following the classification into therapeutic areas. (C) Analysis of the SB claim per therapeutic area regarding the authorized satisfactory methods of treatment, either other therapies or other ATMPs. Total number of applications, *n* = 114.

Fifty-four percent of the ODs included in our analysis contained a discussion on SB, where non-clinical and/or clinical data were used to support the claim of either a CRA or a MCPC. No differentiation between the type of supportive data (clinical or non-clinical) was recorded when the data from the summary reports were collected. Almost all were accepted based on the assumption of a CRA, and only one had a claim of both CRA and MCPC. Only one application was withdrawn during the regulatory evaluation and was noted in our analysis as list of questions (Figure 5A).

The analysis of the claim for SB per therapeutic area highlighted that almost all the areas contained applications in which SB needed to be justified (Figure 5B). Importantly, only products intended for eye disorders needed to justify the assumption of SB over other ATMPs authorized for the rare conditions targeted (Figure 5C). This specific case corresponds to Luxturna, which was authorized in 2018. Other ATMP products, now available on the European market but not when the initial ODs were assessed, were not considered for the SB justification at the time of initial OD since they were not authorized at that moment (the cases of Hemgenix, Roctavian, Zynteglo, and Zolgensma).

## Discussion

The possibility of inserting a corrective gene to replace a mutation in a rare condition offers the opportunity to reverse inexorable disease progression in affected patients.^15^ The COMP has noted an increase in the number of ODs for viral vector-mediated gene therapies targeting non-oncological conditions.^6^ This trend has highlighted that regulatory considerations may differ due to the unique nature of these ATMPs. For regulatory authorities, these new therapies present both opportunities and challenges, potentially altering the evaluation criteria for OD submissions. This retrospective analysis was conducted to determine whether such changes are indeed occurring. Generally, an initial OD application requires a preliminary set of data to support the hypothesis of potential efficacy (MP) and to demonstrate a CRA and/or a MCPC, known as SB, in the designated orphan condition.^7^

Previous research showed that non-clinical *in vivo* data have been used generally in 32% of granted designations.^5^ However, in the case of viral vector-mediated gene therapy, we found that it was used in 95% of the submitted applications, in all the therapeutic areas examined, and most of the studies were conducted in mice. It is reported in the literature that mouse models are the most widely used mammalian animal species in biomedical research, representing more than 60% of the total number of animal species used in the EU.^16^ The potential reasons behind such a high percentage of ODs with this type of data may be linked to the evolution of technologies that generate transgenic mouse models.^17^ Many are widely available for research purposes, making testing the pharmacodynamic and potential effectiveness of a new investigative medicinal product accessible. In addition, a recent publication addressing the orphan concept of MP noted that three main groups of issues associated with non-clinical *in vivo* models relating to “face validity,” “construct validity,” and “predictive validity” should be considered.^13^^,^^14^ In our analysis, mouse models appear to meet these criteria, as they are the model most typically used in successful ODs. Less-often-used models were dog and rat models (Figure 2C), indicating that having models in these species is not that common and that these alternative models may not meet the same validity criteria.

Regarding clinical data, no case histories or compassionate use data were submitted, and clinical data supporting the ODs were derived from clinical trials associated with the development program. This indicates the willingness of the sponsors to move forward into clinical trials to continue product development, rather than limited use through compassionate use programs, with the intention to maximize the effect in the target population. Only a few submissions used exclusively preliminary clinical trial data with the product, and these were submissions for metabolism and hematological disorders.

The greater use of non-clinical *in vivo* data in submissions may indicate the committee’ willingness to accept this level of earlier evidence for gene therapies, and this could be linked to the reported promise that these medicines can address intractable rare diseases.^18^ Seventy-five percent of the non-clinical *in vivo* data submitted used one animal model to support medical plausibility, with 19% using two models and 6% using three or more. This indicates that providing convincing data in one pertinent animal model, especially using disease-related endpoints, might suffice to justify the MP criterion.

Although relevant animal models were used in most of the submissions, in nine applications (as seen in Figure 2A), that was not the case. Arguments put forward by the sponsors to justify the use of non-relevant models were varied. In applications where wild-type animals were used (such as non-human primates [NHPs] and ferrets) the purpose of the study was to demonstrate the expression of the construct and the effect over physiological properties otherwise impaired in the condition. In other cases, the limitations imposed by the products’ features, route of administration, and mode of action precluded the use of relevant models of the condition (e.g., immunodeficient models allowing the use of modified human cells). In some instances, the absence of models mimicking all aspects of the studied disease or presenting early mortality hampered the possibility of testing any active substance, and a different, more suitable model had to be used. The acceptability of available models, be they transgenic or other, was noted to translate into a higher acceptability of the data submitted for these conditions. This offered a higher probability of obtaining an OD, opening the possibility of earlier consultation with regulatory agencies using mechanisms such as protocol assistance to address questions associated with product development.

Thirty-eight percent of the cases used a surrogate product instead of the finished human product. This was justified due to the inability of the latter to test for effectiveness in a non-clinical *in vivo* model. This occurred in three immunodeficiency disorders applications. In other therapeutic areas, such as lysosomal disorders for example, species-specific constructs, like mouse-specific cDNA, were used to overcome cross-species limitations. Given the increasing number of applications containing recombinant or new adeno-associated virus serotypes,^19^ the use of a different viral vector serotype for the assays conducted in animals could explain the observed high frequency of surrogate products used. In other cases, earlier versions of the vector were used in the preliminary non-clinical studies (as an example, containing antibiotic resistance sequences or a different promoter) and differ slightly from the clinical candidate.^20^ As supportive data when non-relevant species and surrogate products were used, either patient-derived cells (in 14 applications) or NHPs (9 applications) (Figure 2E) were used to provide additional evidence in support of the mechanism of action and the long-term expression of the encoded protein.

Around 50% of the ODs granted had non-clinical *in vivo* data with a surrogate endpoint such as a biomarker due to the nature of the condition. This type of endpoint was accepted in submissions in almost all therapeutic areas and often linked to the acceptance by the COMP that functional endpoints were difficult to measure in the target condition. Combinations that involved both surrogate and functional endpoints were the second most noted and covered the therapeutic areas that were more frequently targeted with this type of product. The more complex combination of a triple endpoint comprising surrogate, functional, and survival data were submitted in three treatment areas: musculoskeletal, nervous system, and lysosomal disorders. Models exist where there is a good understanding of functionality and survival of these conditions. Survival measures are of particular interest as many rare conditions are associated with reduced life expectancy, and accordingly, survival outcomes are of great importance for the assessment of OD. Of note, our analysis linked this to lysosomal, hematological, nervous system, metabolism, and musculoskeletal disorders, which correspond to collectively 78 of 114 applications. Such data are considered to be highly supportive for MP and SB by the committee since the aim in many of these conditions is to improve patients’ life expectancy.

A limited number of submissions, namely 13% (15 of 114 submissions), had clinical data. Of these, only 4% had exclusively clinical data, and the remaining 9% had a combination of clinical and non-clinical *in vivo* studies. It was noted in earlier papers that clinical data would be present in around two-thirds of the applications for an OD.^5^ The reason for this difference with gene therapy medicines could be linked to some of the difficulties of obtaining preliminary clinical data due to recruitment concerns associated with very rare conditions.^21^ Most of these submissions had studies with 10 or fewer patients. Only five had more than 10 patients. It has been reported in the literature that conducting clinical trials with gene therapies in rare diseases offers many challenges due to the small number of patients and, in some instances, the coexistence of several new innovative therapies that are competing for these few patients.^22^^,^^23^^,^^24^

SB holds a special place in Europe as it is what makes the EU OD different from other regulatory regions with similar legislation. It attempts to qualify the potential added benefit of a new investigative medicinal product within the recognized standard of care, which generally is associated with authorized medicines but can include well-established satisfactory methods of treatment. In the present analysis, we found that 54% of applications had to address SB, while 46% involved conditions for which there were no authorized satisfactory methods of treatment. Generally, the COMP has noted that in the overall picture of ODs granted, about 66%–70% will have an SB section, while the remainder had no SB to assess as no authorized satisfactory methods of treatment exist.^12^ The higher percentage of gene therapy OD submissions without the need to show SB indicates that many of these innovative therapies are targeting conditions without any treatment available.

In the remaining 54% of cases, SB was based primarily on a CRA that is defined in the legislation as improved efficacy, better safety profile, or better tolerability.^11^ Ninety-nine percent of gene therapy submissions had CRA as the basis for SB, which is significantly higher than that seen in general by the COMP, and this is in the range of around 75%.^10^ The greater use of a CRA claim for gene therapies might be directly linked to the possibility of an early intervention in the disease course and the promise of sustained expression of the encoded protein. Both scenarios can be modeled in relevant non-clinical *in vivo* models of the disease, and the preliminary data used support this claim.

In conclusion, gene therapy development is complex and has its own particularities regarding non-clinical *in vivo* models used, as well as the choice of surrogate endpoints and its translation to clinical development. To encourage these types of therapies that hold promise as curative treatments, acceptance of this level of evidence when applying for an OD is justified, particularly in disease areas where authorized medicines are not available.

We hope that this paper will inform drug developers in this space how to work on a greater chance for successful OD and about early supportive mechanisms such as the pre-licensing incentive protocol assistance.

## Materials and methods

Initial OD summary reports endorsed by the COMP between 2016 and 2021 were extracted from the corresponding EMA databases and classified as previously described.^4^ The total number of applications submitted that met the criteria of “viral vector-mediated gene therapy for non-oncological conditions” was 114. Data extraction focused on two sections of the report, namely MP and SB.

### MP

Data on MP were categorized into *in vitro* (cells), *ex vivo* (whole tissue), non-clinical *in vivo*, and clinical. Endpoints used to support the MP were classified into three major categories: surrogate, functional, and survival. A surrogate endpoint generally refers to a biomarker or indirect pharmacodynamic measure of efficacy, a functional endpoint refers to a clinical symptom associated with the condition such as muscle function, and survival is associated with increase in life as compared to that expected in the condition.

For non-clinical *in vivo* models, the type of animal species and relevance to the condition were recorded, as well as the number of models used. The cases where a surrogate product was used in the non-clinical *in vivo* setting were also noted. We defined a surrogate product as a substitute product used only when the intended one for OD was not compatible with the type of assay (e.g., human-specific viral serotype not transducing mouse cells), or when certain changes to its design are planned during development with a view to maximize the efficacy/safety profile (e.g., changes in non-coding regions or removal of tags).^20^ The pharmacological effect is expected to be at least the same (similar) between both surrogate and final product, and the type of endpoints measured in the non-clinical and clinical setting should measure the same outcomes.

For the ODs where MP was supported by clinical data, the following was recorded into the database: the type of endpoint(s) collected, the number of patients recruited, and the use of surrogate endpoints.

### SB

Data collected for SB were classified with reference to the grounds of the respective COMP opinions into the following broad categories: no SB or SB applicable. In the cases where the demonstration of SB was a requirement, data were further subdivided into SB with CRA, SB with MCPC, or SB with CRA and MCPC at the same time.

## Data and code availability

This paper does not report original code.

## Acknowledgments

No funding was received for this work. The authors wish to thank all EMA and COMP colleagues who indirectly contributed to this manuscript.

## Author contributions

All authors contributed to the project design and critically reviewed the final manuscript. G.M.P. conducted the classification of the products, data extraction, data cleaning, and analysis and wrote the paper. S.M. conducted the identification and classification of the products and the data cleaning and analysis and wrote the paper. T.P.-B. conducted the identification of the products, data cleaning, and data analysis. F.N.-W., E.C., D.M.D., E.M., D.M., and D.V. extracted data from the reports. K.L. helped to draft the manuscript. A.M. and V.S.-B. conceived the project, conducted data extraction, and helped to draft the manuscript.

## Declaration of interests

The views expressed in this article are the personal views of the author(s) and may not be understood or quoted as being made on behalf of or reflecting the position of the regulatory agency/agencies or organizations with which the author(s) is/are employed/affiliated. The authors declare no competing interests.
